# Severe lower respiratory tract infection in infants and toddlers from a non-affluent population: viral etiology and co-detection as risk factors

**DOI:** 10.1186/1471-2334-13-41

**Published:** 2013-01-25

**Authors:** Emerson Rodrigues da Silva, Márcio Condessa Paulo Pitrez, Eurico Arruda, Rita Mattiello, Edgar E Sarria, Flávia Escremim de Paula, José Luis Proença-Modena, Luana Sella Delcaro, Otávio Cintra, Marcus H Jones, José Dirceu Ribeiro, Renato T Stein

**Affiliations:** 1 Universidade de Caxias do Sul, Caxias do Sul, Brazil; 2 Pontifícia Universidade Católica do Rio Grande do Sul, Porto Alegre, Brazil; 3 Universidade de São Paulo, Ribeirão Preto, Brazil; 4 Universidade Estadual de Campinas, Campinas, Brazil; 5 Pediatric Respirology, Department of Pediatrics, PUCRS, Av. Ipiranga, 6690, IPB-PUCRS, Porto Alegre, Brazil

**Keywords:** Respiratory tract infections, Respiratory syncytial virus, Human rhinovirus, Infants, Coinfection

## Abstract

**Background:**

Lower respiratory tract infection (LRTI) is a major cause of pediatric morbidity and mortality, especially among non-affluent communities. In this study we determine the impact of respiratory viruses and how viral co-detections/infections can affect clinical LRTI severity in children in a hospital setting.

**Methods:**

Patients younger than 3 years of age admitted to a tertiary hospital in Brazil during the months of high prevalence of respiratory viruses had samples collected from nasopharyngeal aspiration. These samples were tested for 13 different respiratory viruses through real-time PCR (rt-PCR). Patients were followed during hospitalization, and clinical data and population characteristics were collected during that period and at discharge to evaluate severity markers, especially length of hospital stay and oxygen use. Univariate regression analyses identified potential risk factors and multivariate logistic regressions were used to determine the impact of specific viral detections as well as viral co-detections in relation to clinical outcomes.

**Results:**

We analyzed 260 episodes of LRTI with a viral detection rate of 85% (n = 222). Co-detection was observed in 65% of all virus-positive episodes. The most prevalent virus was Respiratory Syncytial Virus (RSV) (54%), followed by Human Metapneumovirus (hMPV) (32%) and Human Rhinovirus (HRV) (21%). In the multivariate models, infants with co-detection of HRV + RSV stayed 4.5 extra days (p = 0.004), when compared to infants without the co-detection. The same trends were observed for the outcome of days of supplemental oxygen use.

**Conclusions:**

Although RSV remains as the main cause of LRTI in infants our study indicates an increase in the length of hospital stay and oxygen use in infants with HRV detected by RT-PCR compared to those without HRV. Moreover, one can speculate that when HRV is detected simultaneously with RSV there is an additive effect that may be reflected in more severe clinical outcome. Also, our study identified a significant number of children infected by recently identified viruses, such as hMPV and Human Bocavirus (HBov), and this is a novel finding for poor communities from developing countries.

## Background

Lower respiratory tract infections (LRTI) represent an important public health burden in the first years of life accounting for approximately one fifth of all deaths in children below five years of age, especially in developing countries [1]. The specific role of newly identified viruses on LRTIs, like Human Metapneumovirus (hMPV), has been studied in recent years [2]. However, its impact among non-affluent populations has been scarcely evaluated. In such locales, infants with respiratory syncytial virus (RSV)-associated LRTIs present a three times greater risk of a fatal event, when compared to their peers in developed countries [3].

Although RSV is well recognized as the main agent associated with severe LRTIs, recent data indicate that other viruses may play a significant role in these clinical outcomes. Human rhinovirus (HRV) seems to be of particular interest, as the most prevalent virus in respiratory illnesses even in the first years of life [4,5], being associated with severe acute bronchiolitis, especially among children of atopic parents [6]. Moreover, a recent study showed that, in a population of preterm infants, HRV was the most prevalent agent associated with severe bronchiolitis [7]. Also of interest is the fact that wheeze-related HRV infection in the first year of life is associated with an increased risk for developing asthma later in life [8], and that this effect was greater than the observed in relation to RSV [9].

The impact on severity of early life respiratory infections may be also affected by viral co-detections diagnosed through sensitive PCR analyses. Some studies have shown a positive association between viral co-detection and worse clinical outcomes [10,11], while others have failed to show results in the same direction [12-14].

The aims of our study were to determine the current impact of newly identified viruses on the severity of LRTI in infants seen in the emergency room and pediatric wards from a tertiary hospital in a developing country, and how specific viruses alone or in co-detections increased the degree of clinical severity of disease.

## Methods

### Subjects and study design

Infants and toddlers younger than three years of age, with a diagnosis of LRTI, admitted to the emergency room (ER) or pediatric wards of a tertiary hospital in Porto Alegre, southern Brazil, were recruited for this study, during the months of greatest prevalence for acute pediatric respiratory viral illnesses (i.e., from April to November) in 2007 [4,15]. The great majority of patients seen in this particular setting come from low-income families, with health coverage provided by the Brazilian free-access public health system.

LRTI was defined by the presence of signs and symptoms of an acute respiratory infection (cough, nasal discharge, oropharyngeal hyperemia, with or without fever), and lower respiratory signs (tachypnea, retractions, prolonged expiratory time, or crackles/wheezing on auscultation). Chest radiographs were taken only at medical assistant discretion, to avoid unnecessary X-ray exposure, and thus were not used for diagnostic purposes. Children who were admitted in the ER with signs and symptoms of a LRTI for at least 6 hours were considered eligible, once symptoms had started within the previous 5 days. Patients with other co-morbidities such as neuromuscular diseases, previous cardiopulmonary disorders, immunodeficiencies, or important congenital anomalies were excluded. We also excluded patients with a hospitalization due to LRTI in the previous 30 days. Bacterial pneumonia was excluded by clinical presentation and chest X-rays findings.

Within the first 24 hours of hospitalization, medical information was collected from parents or guardians through a standardized questionnaire. Data regarding clinical conditions at admission, vital signs, and signs of respiratory distress were obtained from the medical charts. Information on use of medications, clinical course of the disease until discharge, use of supplemental oxygen, and length of hospital stay were prospectively collected. These two latter variables were used as the main clinical outcomes, serving as surrogates for clinical severity. Supplemental oxygen was withdrawn when pulse oximetry was equal or greater than 94% in room air for at least 6 hours, as this is the standard clinical procedure in the hospital. Sample size was estimated based on few previous similar studies, since data analyzing the association between viral co-detection and our main outcomes was scarce at the time this project was planned [16-18].

### Nasopharyngeal sample collection

Nasopharyngeal aspiration with a standardized technique using vacuum and a sterile collector were performed in all children within the first 48 hours of admission. Samples were immediately split into aliquots, including one in TRIzol^®^, and stored at −80°C, until shipment to the Laboratory of Viral Pathogenesis, at the University of São Paulo School of Medicine, Ribeirão Preto.

### Viral detection by individual real-time RT-PCR

To isolate RNA from nasal aspirates, 250 μL were extracted according to the manufacturer’s protocol (Invitrogen, Carlsbard, USA). DNA was extracted from a sample of 200 μL of nasal aspirate using the Wizard Genomic DNA^®^ purification kit, following manufacturer’s instructions (Promega, Madison, USA). The detection of viruses was done by Real Time PCR, using the Taqman System^®^ (Applied Biosystems, New Jersey, USA), with specific primers and probes in a thermal cycler (7300 Real Time PCR system^®^ - Applied Biosystems). Real Time PCR reactions for cellular gene (β-actin) were also performed for internal quality control.

For viruses with a RNA genome (i.e., HRV, Influenza Virus-A [FLUAV], human parainfluenza virus [HPIV], RSV, hMPV and human coronavirus [HCoV]), the transcription into cDNA was done with reverse transcriptase (high capacity cDNA reverse transcription kit^®^, Applied Biosystems), using 1 μg from the extracted RNA, according to the manufacturers’ protocol. The qPCR assays were performed using 3 μL of the DNA extraction or cDNA (approximately 150 ng), 0.33 pmoles for primers, 0.17 pmoles for probes and 7.5 μL of master mix of TaqMan^®^ (Applied Biosystems). Amplifications were performed with 45 cycles of denaturation at 95°C for 15 seconds and annealing-extension at 60°C for one minute, except for hMPV, when annealing was done at 55°C for 30 seconds, and extension at 60°C for one minute.

### Statistical analysis

Demographics were summarized as mean or median and range according to their distribution. Characteristics among groups were compared, accordingly, using two-sample t-test, Mann–Whitney, Chi-square or Fisher’s exact test.

Generalized linear models (Tweedie model with Identity link function) were used to analyze the relationships between main outcomes (length of stay in the hospital and time in use of supplemental oxygen) and the predictor variables (virus detection [yes/no], sex, age, prematurity (i.e. <37 weeks of gestation), maternal smoking during pregnancy, family asthma). All variables with a significance of p < 0.15 were considered in the univariate models and those with significance of p < 0.05 in the multivariate analysis. All analyses were performed using SPSS v.18 (SPSS Inc, Chicago, IL).

This study was approved by the local Institutional Ethics Committees (06/03467 - Pontifícia Universidade Católica do Rio Grande do Sul and 4856/2004 – Universidade de São Paulo). Parents or legal guardians read and signed an informed consent approved for this study.

## Results

Two hundred and sixty patients were enrolled in the study. Characteristics of the patients recruited are presented in Table 1. In the whole sample there were more boys than girls, almost half of children were younger than six months, 24% of all children were born premature, 29% had been exposed to tobacco during gestation, and more than a half had a family history of asthma (parents or siblings). Overall, median hospital length was 6 days and median supplemental oxygen requirement was 5 days.

**Table 1:** Characteristics and outcomes of the population included in the study (260 children)


Sex, female, n (%)	103 (40)
Age, months, median (range)	5 (1-35)
Age, <6 months, n (%)	128 (49)
Maternal smoking during pregnancy, n (%)	74 (29)
Prematurity, n (%)	62 (24)
Family history of asthma*, n (%)	149 (58)
LOS^+^, days, median (range)	4 (2-8)
Use of oxygen, days, median (range)	3 (1-6)

^+ ^LOS: length of hospital stay; * for parents and/or siblings.

The presence of viruses was detected by PCR in 222 (85%) of all LRTI episodes. Co-detection was present in 146/260 (56%). The frequencies of viral detection and co-detection are shown in Table 2. The most common single infection was related to RSV (54%), followed by hMPV (32%), and HRV (21%).

**Table 2:** Viral detection by RT-PCR, among 260 children

**Overall prevalence of viruses**	**n (%)**
No vírus detected	38 (14.1)
RSV	139 (53.5)
hMPV	84 (32.3)
HRV	54 (20.8)
HBov	27 (10.4)
Influenza (A or B)	33 (12.7)
Para-influenza (1 or 2)	17 (6.5)
Adenovirus	17 (6.5)
Coronavirus	3 (1.2)
Co-detections	n (%)
RSV + HRV	22 (8.5)
RSV + HMPV	37 (14.2)
RSV + Others*	26 (10.0)
HRV + Others**	29 (11.2)
HMPV + Others***	16 (6.1)
HMPV + Others***	16 (6.1)

RSV: Respiratory sincytial virus; HMPV: Human metapneumovirus; HRV: Human rhinovirus.

* Bocavirus + influenza A or B.

** HMPV + influenza A or B.

*** Bocavirus + influenza A or B.

**Figure 1:**
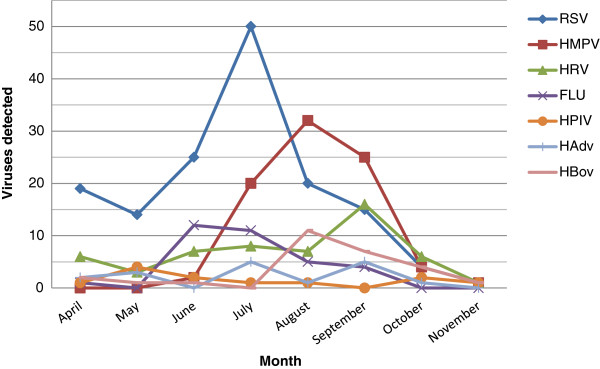
Monthly numbers of samples positive for the different viruses from the subjects studied.

During the surveyed time RSV has shown an incidence peak in the beginning of the cold season (i.e. from april to November) in the southern hemisphere, followed in late winter by peaks of hMPV, HRV, and HBov (Figure 1). Other viruses such as HPIV and Human Adenovirus (HAdv) showed low but constant rates throughout the season.

In the univariate analyses, length of hospital stay and need of supplemental oxygen were significantly associated with age (≤6 months), maternal smoking during pregnancy and with family history of asthma (parents and/or siblings) (Table 3). Infants younger than 6 months of age stayed in hospital 3.8 days longer than older infants (p < 0.001), and those with a family history of asthma stayed 2.4 days longer than those without a family history of asthma (p < 001). Also, infants 6 months of age or younger needed supplemental oxygen for an extra 3.8 days, when compared to older infants/children. A similar finding was observed for children with a family history of asthma, who required 2.4 extra days of oxygen compared to those without the family history of asthma (Table 3). Other risk factors, such as breastfeeding, indoor smoking, current parental smoking, siblings, and overcrowding were not significantly associated with neither of the main outcomes.

**Table 3:** Univariate analyses of risk factors for length of hospital stay and days with supplemental oxygen (n = 260)

	**Length of hospital stay**			**Days with supplemental oxygen**		
	β*	**(95% CI)**	***p***	β*	**(95% CI)**	***p***
Sex, female	0.4	(-0.9 - 1.7)	0.553	0.5	(-0.6 - 1.7)	0.372
Age, <6 months	3.8	(2.7 - 5)	<0.001	3.8	(2.8 - 4.9)	<0.001
Prematurity	0.4	(-1.0 - 1.9)	0.570	0.4	(-1.0 - 1.7)	0.605
Maternal smoking during pregnancy	1.2	(-0.2 - 2.6)	0.106	1.3	(-0.02 - 2.7)	0.054
Family Hx asthma	2.4	(1.2 - 3.6)	<0.001	2.4	(1.3 - 3.5)	<0.001

Hx: history; * number of extra days, compared to negative counterparts.

Infants with positive PCR for HRV alone as well as those co-detected with RSV and HRV also had significantly longer hospital stays (3.2 days, p = 0.001; and 5.5 days, p = 0.002, respectively) than those with other detected viruses. Extended time in use of supplemental oxygen was also associated with HRV (2.8 days, p = 0.002) and RSV (3.7 days, p = 0.013), but also with Influenza virus A or B (2.2 days, p = 0.042), when compared to those with other viruses in single or in co-detection.

**Figure 2:**
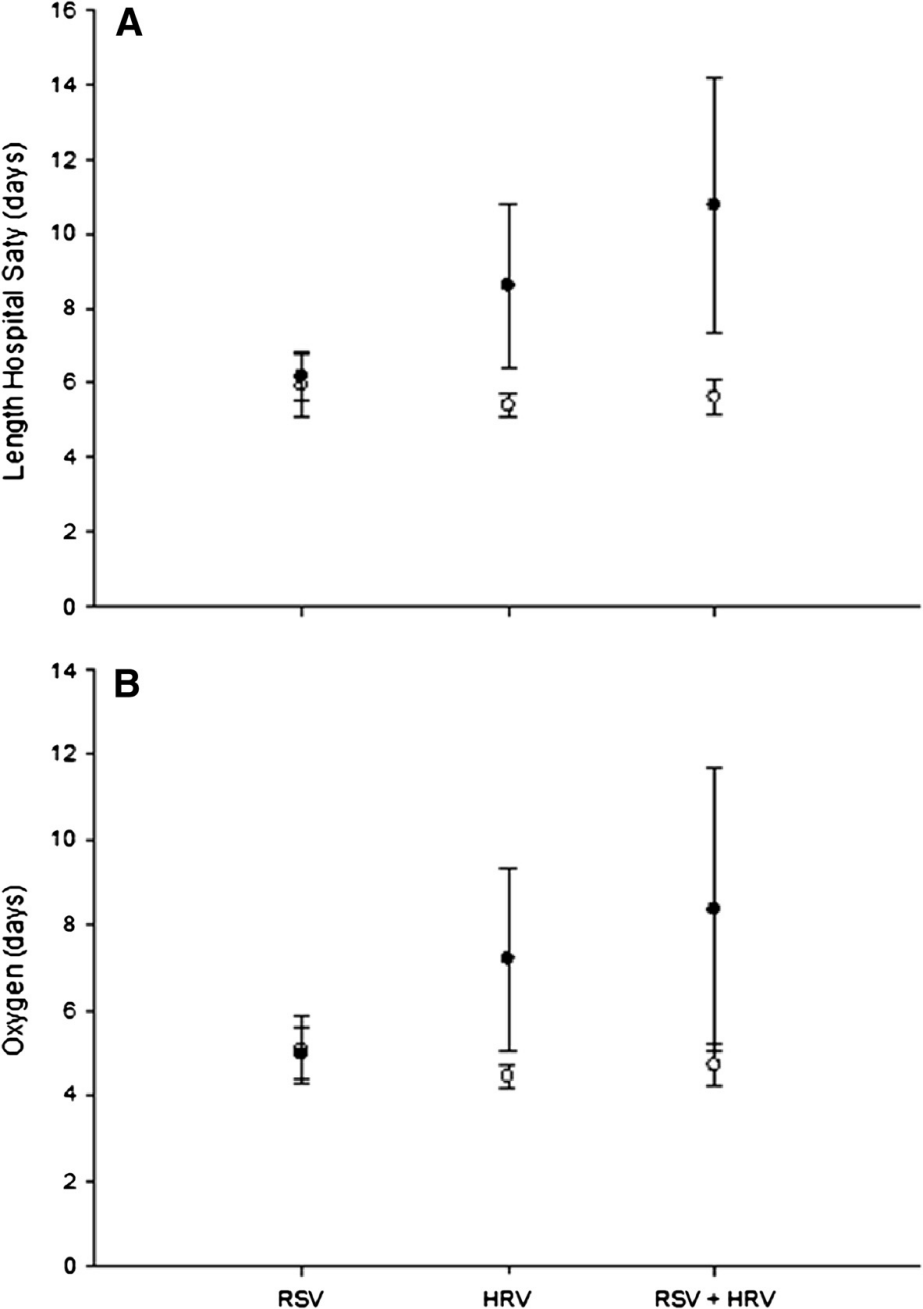
**Comparison of RSV, HRV and RSV plus HRV in regards to: (A) length of hospital stay and (B) days of supplemental oxygen use. **Black circles: group of infants ≤ 6 months of age; white circles: group of infants > 6 months of age. The groups of HRV and RSV + HRV were significantly different (p < 0.05), when compared by age. Values expressed as mean ± SD of days.

Infants with HRV-LRTIs stayed an extra 2.2 days in hospital (p = 0.011), for a total of 7.7 (95% CI: 6.1-9.3) days when compared to those with other infections, after adjusting for potential confounding variables (Table 4). Table 5 shows that infants with combined HRV and RSV positive PCR in the same samples stayed 4.5 extra days (p = 0.004) than those without HRV and RSV in these adjusted models (that included sex, age ≤ 6 versus >6 months, prematurity, family history of asthma and maternal smoking during pregnancy), (95% CI: 7.0-13.0) days. Time in use of supplemental oxygen followed the same association trends. Infants with proven RSV infections needed 4.75 (95% CI: 3.97-5.53) extra days of oxygen, while those with HRV used supplemental oxygen for 1.4 extra days, and those with RSV and HRV co-detection for 2.2 days, when compared to infants presenting positive PCRs for other viruses alone or in combinations. Figure 2 illustrates the association between HRV and RSV + HRV co-detection with increased length of hospital stay and oxygen use, and this effect is especially significant for infants younger than 6 months of age. Influenza viruses were not associated with longer use of supplemental oxygen when controlling for the demographic variables.

**Table 4:** Multivariate analysis for length of hospital stay (dependent variable), and HRV adjusting other risk factors

	**Yes**		**No**		β*	**95% CI**	***P***
	**Mean**	**(95% CI)**	**Mean**	**(95% CI)**			
HRV	7.7	(6.1 - 9.3)	5.5	(4.7 - 6.2)	2.2	(0.5 - 3.9)	0.011
Sex (female)	6.8	(5.6 - 8.0)	6.3	(5.4 - 7.3)	0.4	(-0.6 - 1.5)	0.419
Age <6 months	8.2	(7.0 - 9.3)	5.0	(3.9 - 6.0)	3.2	(2.0 - 4.3)	<0.001
Family Hx of asthma	7.2	(6.2 - 8.2)	6.0	(4.8 - 7.1)	1.2	(0.1 - 2.3)	0.035
Prematurity	6.7	(5.4 - 7.9)	6.5	(5.5 - 7.4)	-0.2	(-1.4 - 1.0)	0.749
Maternal smoking	6.8	(5.6 - 8.2)	6.3	(5.3 - 7.2)	0.6	(-0.6 - 1.8)	0.354

Hx: history; * number of extra days in hospital, compared to negative counterparts.

**Table 5:** Multivariate analysis for length of hospital stay (dependent variable) and HRV + RSV (co-detection), adjusting for other risk factors

	**Yes**		**No**		β*	**(95% CI)**	***P***
	**Mean**	**(95% CI)**	**Mean**	**(95% CI)**			
RSV + HRV	10.0	(7.0 - 13.0)	5.5	(4.8 - 6.3)	4.5	(1.4 - 7.5)	0.004
Age, <6 months	9.4	(7.7 - 11.1)	6.1	(4.6 - 7.7)	3.3	(2.2 - 4.5)	<0.001
Sex (female)	7.9	(6.2 - 9.6)	7.6	(6.0 - 9.2)	0.3	(-0.7 - 1.4)	0.517
Family Hx of asthma	8.3	(6.7 - 9.9)	7.2	(5.5 - 8.9)	1.1	(0.04 - 2.2)	0.043
Prematurity	7.8	(6.1 - 9.5)	7.7	(6.1 - 9.4)	-0.04	(-1.2 - 1.2)	0.952
Maternal smoking	8.1	(6.3 - 9.9)	7.4	(5.9 - 8.9)	0.7	(-0.5 - 2.0)	0.243

Hx: History; * number of extra days in hospital, compared to negative counterparts.

## Discussion

Our results suggest that infants with severe LRTI and positive PCR for HRV, alone or in co-detection with RSV, stayed hospitalized longer periods and utilized more supplemental oxygen, when compared to children infected by other viruses, including those with RSV-alone. Our data also reinforce previous findings that identified RSV as the major agent associated with severe LRTIs among children in a hospital setting, in populations of low socio-economic status, where other environmental and social variables potentially play a role [16,17]. Infants younger than six months and those with a family history of asthma/recurrent wheeze are also at greater risk for disease severity.

RSV was the most frequently detected virus, accounting for a high burden of LRTIs in our population. Although it is not possible to establish an unequivocal correlation between LRTIs and upper airway viral detection, the finding of RSV in over 50% of hospitalized children in our study strongly suggests that its impact is still indeed very high in this region, regardless of the presence of newly identified viruses. These results are in accordance with recently published studies in Brazil, which also identified RSV as the main agent responsible for severe LRTI, especially in a hospital setting. Nascimento and coworkers have shown an overall viral detection rate of 93% from nasopharyngeal samples in a small group of children below 2 years of age, and reported RSV as the most prevalent virus (63.6%) [19]. Another study aiming to investigate the role of HBov and hMPV in LRTIs in southern Brazil also showed similar results (at least one positive virus in 90% of the samples and RSV positive in 49.3%) [20]. The high detection rate of RSV in children with LRTI in a hospital setting such as ours is consistent with most studies worldwide and the burden due to viral respiratory disease seems as high in these locales as they are in more developed countries. A recent analysis of children admitted into the hospital due to acute bronchiolitis in Texas, USA, has shown a steady increase in admissions over a 5-year period and this has been credited mostly to RSV [21]. This increasing role for severe viral LRTIs, observed also in other studies, is probably explained by a series of complex environmental and social changes that seem to affect how viruses spread in communities.

Some studies have reported different clinical outcomes for specific viruses causing LRTIs, especially in the presence of co-detections, such as with RSV and HBov [22,23], while others did not reproduce such findings [17,24,25] and these associations remain unclear. A lack of association between an overall finding of any viral co-detection and LRTI severity was reported in studies performed in non-affluent countries [2,12,26], as well as in developed countries [13,14]. In our study, patients detected with HRV alone and RSV + HRV presented increased length of hospitalization and increased time of supplemental oxygen use. Papadopoulos et al. has shown a five-fold increase in clinical severity in infants with acute bronchiolitis due to HRV, compared to those infected only with RSV. Compared to those with positive RSV samples without HRV co-detection, infants with HRV were older, had lower birth weights and were hospitalized earlier [16]. Some explanations for apparently contradictory findings in a myriad of studies could be attributed to the lack of uniform criteria for subject inclusion and standardized statistical analysis [14]. Other plausible explanations are the natural variation in HRV prevalence in different seasons and possible variations in the prevalence of type C HRV. This agent is associated with more severe disease and was already described as a major cause of LRTI in infants from non-affluent countries [27,28]. Unfortunately, in our study, we were not able to determine the prevalence of HRV subtypes, and this stands as an interesting subject for further research.

The association of HRV (alone or with RSV) with LRTI severity and atopy has not been widely studied. The relationship between persistent wheezing at 3 years and at 6 years versus relevant HRV infection in early life is well established [9,29], but there are few studies looking at these relationships in the first year of life. A recent study suggests that the relationship between HRV in early life LRTI and subsequent recurrent wheeze/asthma is dependent on allergic sensitization, which seems to precede the viral insult in a causal model [30]. This association we have found between HRV with increased severity (using the surrogates of length of hospital stay and days in supplemental oxygen) is a major finding. Hence, the association between HRV infection, increased severity and atopy remains to be better clarified.

It has already been shown that HRV is able to reduce cell proliferation and decreases the self-repair capacity of bronchial epithelial cells [31]. Therefore, our data may suggest that in certain subsets of patients the burden of HRV in acute LRTI should be considered distinct from that of other viruses. Another plausible explanation for our findings could be the possibility of HRV persistence in the airways leading to an “over detection” of the virus, simultaneously with those infected only by RSV [32]. In our study this hypothesis seems improbable since we detected a clear worsening in the clinical markers in patients with RSV that were also detected with HRV.

Another interesting finding of our study was the high prevalence of newly described viruses. hMPV was detected in almost one third of all episodes, but did not seem to affect the main outcomes studied here, in the way RSV and HRV have done. While the recognition of the impact of human hMPV is increasing, its prevalence is still probably underestimated in clinical practice, since laboratory testing has become widely available only in recent years [18,24,33]. HBov was also detected in a large number of nasal samples and it was very frequently associated (co-detected) with other agents. In our study, only 2/27 patients with HBov had this virus detected as single agent. The overall HBov detection rate was higher in our data compared to previous studies [34,35], and this may be explained again by natural seasonal variations. It is also interesting to notice the seasonal pattern of both hMPV and HBov, which present their peak prevalence rates in late winter, right after the RSV peak, which occurs earlier in winter.

The main limitation of our study is the lack of surveillance in consecutive years, which could have biased results in case of an outbreak of one specific virus in a given year. Our eight months of viral surveillance could potentially have failed to detect any atypical outbreak, which did not seem likely to have occurred. Also, reliable tests capable of ruling out bacterial co-detection were not available at the time of the study. This could have underestimated the burden of bacteria in our sample and the co-detection of viruses and bacteria remains an interesting issue for further studies.

## Conclusion

In our study, RSV was the most prevalent viral agent in hospitalized patients with LRTI and the co-detection of HRV in patients with RSV infection increased hospital stay and days in use of supplemental oxygen. Interestingly, even in developing countries, the role of recently discovered viruses needs to be further studied in order to identify novel risk factors of susceptibility/severity, and new treatment targets for these agents. We also highlight the role of HRV as an important risk factor for severe LRTI, particularly when simultaneously associated with RSV, which strongly suggests that co-detection may also mean co-infection, since the combination of the two agents seem to affect clinical outcomes. Longitudinal studies with control groups are necessary to confirm these results in populations at greater risk for severe respiratory disease.

## Abbreviations

LRTI: Lower respiratory tract infection; RT-PCR: Real-time polymerase chain reaction; RSV: Respiratory syncytial virus; hMPV: Human metapneumovirus; HRV: Human rhinovirus; HBov: Human bocavirus; ER: Emergency room; FLUA: Influenza virus A; HPIV: Human parainfluenza virus; HCov: Human coronavirus; HAdv: Human adenovirus

## Competing interests

This study was supported by Abbott Laboratórios do Brasil Ltda (academic grant), from an unrestricted investigator-generated proposal.

## Authors’ contributions

ERS and RS participated in all steps of the study. PMP participated in the design of the study, in data analysis and manuscript review. E.A. participated in study design and in the RT-PCR essays. FEP, LSD and JLPM participated in the RT-PCR essays. RM and EES performed the statistical analysis and have reviewed the manuscript. MHJ participated in the data collection. OS and JDR participated in the conception and design of the study. All authors read and approved the final manuscript.

## Pre-publication history

The pre-publication history for this paper can be accessed here:

http://www.biomedcentral.com/1471-2334/13/41/prepub

## References

[B1] Global action plan for prevention and control of pneumonia (GAPP) http://www.who.int/maternal_child_adolescent/documents/9789241596336/en/10.2471/BLT.08.053348 PMC2647444 18545727

[B2] Freymuth F Vabret A Cuvillon-Nimal D Simon S Dina J Legrand L Gouarin S Petitjean J Eckart P Brouard J Comparison of multiplex PCR assays and conventional techniques for the diagnostic of respiratory virus infections in children admitted to hospital with an acute respiratory illness J Med Virol 2006 78 11 1498 1504 10.1002/jmv.20725 16998894 PMC7159369

[B3] Nair H Nokes DJ Gessner BD Dherani M Madhi SA Singleton RJ O’Brien KL Roca A Wright PF Bruce N Global burden of acute lower respiratory infections due to respiratory syncytial virus in young children: a systematic review and meta-analysis Lancet 2010 375 9725 1545 1555 10.1016/S0140-6736(10)60206-1 20399493 PMC2864404

[B4] Pitrez PM Stein RT Stuermer L Macedo IS Schmitt VM Jones MH Arruda E [Rhinovirus and acute bronchiolitis in young infants] J Pediatr (Rio J) 2005 81 5 417 420 10.2223/JPED.1394 16247546

[B5] van der Zalm MM Uiterwaal CS Wilbrink B de Jong BM Verheij TJ Kimpen JL van der Ent CK Respiratory pathogens in respiratory tract illnesses during the first year of life: a birth cohort study Pediatr Infect Dis J 2009 28 6 472 476 10.1097/INF.0b013e318195e26e 19504730

[B6] Miller EK Williams JV Gebretsadik T Carroll KN Dupont WD Mohamed YA Morin LL Heil L Minton PA Woodward K Host and viral factors associated with severity of human rhinovirus-associated infant respiratory tract illness J Allergy Clin Immunol 2011 127 4 883 891 10.1016/j.jaci.2010.11.041 21269669 PMC3070861

[B7] Miller EK Bugna J Libster R Shepherd BE Scalzo PM Acosta PL Hijano D Reynoso N Batalle JP Coviello S Human rhinoviruses in severe respiratory disease in very low birth weight infants Pediatrics 2012 129 1 e60 e67 10.1542/peds.2011-0583 22201153 PMC3255465

[B8] Jackson DJ Johnston SL The role of viruses in acute exacerbations of asthma J Allergy Clin Immunol 2010 125 6 1178 1187 quiz 1188–1179 10.1016/j.jaci.2010.04.021 20513517 PMC7172767

[B9] Jackson DJ Gangnon RE Evans MD Roberg KA Anderson EL Pappas TE Printz MC Lee WM Shult PA Reisdorf E Wheezing rhinovirus illnesses in early life predict asthma development in high-risk children Am J Respir Crit Care Med 2008 178 7 667 672 10.1164/rccm.200802-309OC 18565953 PMC2556448

[B10] Calvo C Garcia-Garcia ML Blanco C Vazquez MC Frias ME Perez-Brena P Casas I Multiple simultaneous viral infections in infants with acute respiratory tract infections in Spain Journal of clinical virology: the official publication of the Pan American Society for Clinical Virology 2008 42 3 268 272 10.1016/j.jcv.2008.03.012 18455958 PMC7108242

[B11] Richard N Komurian-Pradel F Javouhey E Perret M Rajoharison A Bagnaud A Billaud G Vernet G Lina B Floret D The impact of dual viral infection in infants admitted to a pediatric intensive care unit associated with severe bronchiolitis Pediatr Infect Dis J 2008 27 3 213 217 10.1097/INF.0b013e31815b4935 18277932

[B12] De Paulis M Gilio AE Ferraro AA Ferronato AE do Sacramento PR Botosso VF Oliveira DB Marinheiro JC Harsi CM Durigon EL Severity of viral coinfection in hospitalized infants with respiratory syncytial virus infection J Pediatr (Rio J) 2011 87 4 307 313 21655684 10.2223/JPED.2100

[B13] Marguet C Lubrano M Gueudin M Le Roux P Deschildre A Forget C Couderc L Siret D Donnou MD Bubenheim M In very young infants severity of acute bronchiolitis depends on carried viruses PLoS One 2009 4 2 e4596 10.1371/journal.pone.0004596 19240806 PMC2644758

[B14] Suryadevara M Cummings E Bonville CA Bartholoma N Riddell S Kiska D Rosenberg HF Domachowske JB Viral etiology of acute febrile respiratory illnesses in hospitalized children younger than 24 months Clin Pediatr 2011 50 6 513 517 10.1177/0009922810394834 PMC3417762 21262758

[B15] Thomazelli LM Vieira S Leal AL Sousa TS Oliveira DB Golono MA Gillio AE Stwien KE Erdman DD Durigon EL Surveillance of eight respiratory viruses in clinical samples of pediatric patients in southeast Brazil J Pediatr (Rio J) 2007 83 5 422 428 10.1590/S0021-75572007000600005 17940688

[B16] Papadopoulos NG Moustaki M Tsolia M Bossios A Astra E Prezerakou A Gourgiotis D Kafetzis D Association of rhinovirus infection with increased disease severity in acute bronchiolitis Am J Respir Crit Care Med 2002 165 9 1285 1289 10.1164/rccm.200112-118BC 11991880

[B17] Wilkesmann A Schildgen O Eis-Hubinger AM Geikowski T Glatzel T Lentze MJ Bode U Simon A Human metapneumovirus infections cause similar symptoms and clinical severity as respiratory syncytial virus infections Eur J Pediatr 2006 165 7 467 475 10.1007/s00431-006-0105-4 16607540

[B18] Williams JV Harris PA Tollefson SJ Halburnt-Rush LL Pingsterhaus JM Edwards KM Wright PF Crowe JE Jr Human metapneumovirus and lower respiratory tract disease in otherwise healthy infants and children N Engl J Med 2004 350 5 443 450 10.1056/NEJMoa025472 14749452 PMC1831873

[B19] Nascimento MS Souza AV Ferreira AV Rodrigues JC Abramovici S Silva Filho LV High rate of viral identification and coinfections in infants with acute bronchiolitis Clinics (Sao Paulo) 2010 65 11 1133 1137 10.1590/S1807-59322010001100014 21243286 PMC2999709

[B20] Pilger DA Cantarelli VV Amantea SL Leistner-Segal S Detection of human bocavirus and human metapneumovirus by real-time PCR from patients with respiratory symptoms in Southern Brazil Memorias do Instituto Oswaldo Cruz 2011 106 1 56 60 10.1590/S0074-02762011000100009 21340356

[B21] García CG Bhore R Soriano-Fallas A Trost M Chason R Ramilo O Mejias A Risk Factors in Children Hospitalized With RSV Bronchiolitis Versus Non–RSV Bronchiolitis Pediatrics 2010 126 6 e1453 e1460 10.1542/peds.2010-0507 21098154 PMC3761792

[B22] Midulla F Scagnolari C Bonci E Pierangeli A Antonelli G De Angelis D Berardi R Moretti C Respiratory syncytial virus, human bocavirus and rhinovirus bronchiolitis in infants Arch Dis Child 2010 95 1 35 41 10.1136/adc.2008.153361 19822538

[B23] Moriyama Y Hamada H Okada M Tsuchiya N Maru H Shirato Y Maeda Y Hirose Y Yoshida M Omura Y Distinctive clinical features of human bocavirus in children younger than 2 years Eur J Pediatr 2010 169 9 1087 1092 10.1007/s00431-010-1183-x 20383526 PMC2908446

[B24] Zhang SX Tellier R Zafar R Cheung R Adachi D Richardson SE Comparison of human metapneumovirus infection with respiratory syncytial virus infection in children Pediatr Infect Dis J 2009 28 11 1022 1024 10.1097/INF.0b013e3181aa6853 19730154

[B25] Bezerra PG Britto MC Correia JB Duarte Mdo C Fonceca AM Rose K Hopkins MJ Cuevas LE McNamara PS Viral and atypical bacterial detection in acute respiratory infection in children under five years PLoS One 2011 6 4 e18928 10.1371/journal.pone.0018928 21533115 PMC3078930

[B26] Venter M Lassauniere R Kresfelder TL Westerberg Y Visser A Contribution of common and recently described respiratory viruses to annual hospitalizations in children in South Africa J Med Virol 2011 83 8 1458 1468 10.1002/jmv.22120 21678450 PMC7166348

[B27] Fuji N Suzuki A Lupisan S Sombrero L Galang H Kamigaki T Tamaki R Saito M Aniceto R Olveda R Detection of human rhinovirus C viral genome in blood among children with severe respiratory infections in the Philippines PLoS One 2011 6 11 e27247 10.1371/journal.pone.0027247 22087272 PMC3210775

[B28] Linsuwanon P Payungporn S Samransamruajkit R Posuwan N Makkoch J Theanboonlers A Poovorawan Y High prevalence of human rhinovirus C infection in Thai children with acute lower respiratory tract disease J Infect 2009 59 2 115 121 10.1016/j.jinf.2009.05.009 19556008 PMC7172887

[B29] Lemanske RF Jr Jackson DJ Gangnon RE Evans MD Li Z Shult PA Kirk CJ Reisdorf E Roberg KA Anderson EL Rhinovirus illnesses during infancy predict subsequent childhood wheezing J Allergy Clin Immunol 2005 116 3 571 577 10.1016/j.jaci.2005.06.024 16159626

[B30] Jackson DJ Evans MD Gangnon RE Tisler CJ Pappas TE Lee WM Gern JE Lemanske RF Jr Evidence for a causal relationship between allergic sensitization and rhinovirus wheezing in early life Am J Respir Crit Care Med 2012 185 3 281 285 10.1164/rccm.201104-0660OC 21960534 PMC3297109

[B31] Xatzipsalti M Psarros F Konstantinou G Gaga M Gourgiotis D Saxoni-Papageorgiou P Papadopoulos NG Modulation of the epithelial inflammatory response to rhinovirus in an atopic environment Clinical and experimental allergy: journal of the British Society for Allergy and Clinical Immunology 2008 38 3 466 472 10.1111/j.1365-2222.2007.02906.x 18269670

[B32] Mahony JB Detection of respiratory viruses by molecular methods Clin Microbiol Rev 2008 21 4 716 747 10.1128/CMR.00037-07 18854489 PMC2570148

[B33] van den Hoogen BG de Jong JC Groen J Kuiken T de Groot R Fouchier RA Osterhaus AD A newly discovered human pneumovirus isolated from young children with respiratory tract disease Nat Med 2001 7 6 719 724 10.1038/89098 11385510 PMC7095854

[B34] Arnold JC Singh KK Spector SA Sawyer MH Undiagnosed respiratory viruses in children Pediatrics 2008 121 3 e631 e637 10.1542/peds.2006-3073 18310182

[B35] Zheng LS Yuan XH Xie ZP Jin Y Gao HC Song JR Zhang RF Xu ZQ Hou YD Duan ZJ Human bocavirus infection in young children with acute respiratory tract infection in Lanzhou, China J Med Virol 2010 82 2 282 288 10.1002/jmv.21689 20029808 PMC7166553

